# Characterization of zebrafish (*Danio rerio*) muscle ankyrin repeat proteins reveals their conserved response to endurance exercise

**DOI:** 10.1371/journal.pone.0204312

**Published:** 2018-09-25

**Authors:** Srdjan Boskovic, Rubén Marín-Juez, Jovana Jasnic, Sven Reischauer, Hadil El Sammak, Ana Kojic, Georgine Faulkner, Dragica Radojkovic, Didier Y. R. Stainier, Snezana Kojic

**Affiliations:** 1 Laboratory for Molecular Biology, Institute of Molecular Genetics and Genetic Engineering, University of Belgrade, Belgrade, Serbia; 2 Department of Developmental Genetics, Max Planck Institute for Heart and Lung Research, Bad Nauheim, Germany; 3 Department of Biology, University of Padova, Padova, Italy; University of Maryland Center for Environmental Science, UNITED STATES

## Abstract

Muscle proteins with ankyrin repeats (MARPs) ANKRD1 and ANKRD2 are titin-associated proteins with a putative role as transcriptional co-regulators in striated muscle, involved in the cellular response to mechanical, oxidative and metabolic stress. Since many aspects of the biology of MARPs, particularly exact mechanisms of their action, in striated muscle are still elusive, research in this field will benefit from novel animal model system. Here we investigated the *MARPs* found in zebrafish for protein structure, evolutionary conservation, spatiotemporal expression profiles and response to increased muscle activity. Ankrd1 and Ankrd2 show overall moderate conservation at the protein level, more pronounced in the region of ankyrin repeats, motifs indispensable for their function. The two zebrafish genes, *ankrd1a* and *ankrd1b*, counterparts of mammalian *ANKRD1/Ankrd1*, have different expression profiles during first seven days of development. Mild increase of *ankrd1a* transcript levels was detected at 72 hpf (1.74±0.24 fold increase relative to 24 hpf time point), while *ankrd1b* expression was markedly upregulated from 24 hpf onward and peaked at 72 hpf (92.18±36.95 fold increase relative to 24 hpf time point). Spatially, they exhibited non-overlapping expression patterns during skeletal muscle development in trunk (*ankrd1a*) and tail (*ankrd1b*) somites. Expression of *ankrd2* was barely detectable. Zebrafish *MARPs*, expressed at a relatively low level in adult striated muscle, were found to be responsive to endurance exercise training consisting of two bouts of 3 hours of forced swimming daily, for five consecutive days. Three hours after the last exercise bout, *ankrd1a* expression increased in cardiac muscle (6.19±5.05 fold change), while *ankrd1b* and *ankrd2* were upregulated in skeletal muscle (1.97±1.05 and 1.84±0.58 fold change, respectively). This study provides the foundation to establish zebrafish as a novel *in vivo* model for further investigation of MARPs function in striated muscle.

## Introduction

The MARP family of stress responsive proteins is composed of three members: cardiac ankyrin repeat protein (ANKRD1/CARP), ankyrin repeat domain protein 2 (ANKRD2/ARPP) and diabetes related ankyrin repeat protein (ANKRD23/DARP) [1]. Their expression is mainly localized to cardiac and skeletal muscles, but to a different extent. In mammals, ANKRD1 and ANKRD2 proteins are predominantly expressed in cardiac and skeletal muscles, respectively [2–5], while *DARP* transcript is equally distributed between these tissues [6]. MARPs are implicated in a number of functions, ranging from mechanosensing to modulation of different signaling pathways and transcriptional regulation [1].

In striated muscle these proteins respond to various forms of mainly mechanical stress [7–14] which affect their expression level and cellular distribution. After prolonged stretch ANKRD1 and DARP proteins redistribute to the nucleus of fetal rat cardiac myocytes [15], while *Ankrd2* gene expression is upregulated [10, 16]. Shuttling of ANKRD2 to the nucleus was observed in stressed mouse muscle fibers [17] and myofibers with damaged sarcomeres [18]. Accordingly, it is proposed that MARPs link the myofibrillar stress-related signaling pathways and muscle gene expression via stress-induced relocation from the cytoplasm to the nucleus [15]. ANKRD1 is involved in cardiomyocyte stress-response networks activated by myocardial infarction or pressure overload that leads to hypertrophy and heart failure [19]. It appears that ANKRD1 may have a more general role in mediating stress response, since its level is highly induced during the healing process of skin wounds in mice [20]. Overexpression of human ANKRD1 has been shown to improve several aspects of wound healing, including neoangiogenesis [21]. Apart from mechanical stress, oxidative stress was found to regulate intracellular localization of ANKRD2 [22], causing nuclear shuttling of overexpressed protein. Stress responsiveness of ANKRD2 is tightly related to its role in coordination of myogenic differentiation [23], but little is known about the function of ANKRD2 in mature muscle.

Altered expression of MARPs has been reported in various pathological conditions of the heart and skeletal muscle, indicating their clinical relevance [24–29]. Elevated expression level of *ANKRD1* mRNA and protein was detected in patients with end-stage heart failure [29], as well as in dilated, hypertrophic and arrhythmogenic ventricular cardiomyopathies [28, 30–32]. Several studies have linked mutations in the *ANKRD1* gene with cardiomyopathies [31–34]. ANKRD2 protein expression is altered in various skeletal muscle pathologies [5, 26, 27] and is likely associated with transition of muscle fiber types [16]. Recent findings suggest that ANKRD2 acts as a mediator of the pathological functions of the mutated *LMNA* gene in Emery-Dreifuss muscular dystrophy 2 (EDMD2). It was shown that mutated lamin A sequesters and mislocates ANKRD2 in the nucleus of EDMD2-affected human myotubes [35]. Although murine MARP proteins are not essential for development and function of cardiac and skeletal muscles, MARP triple knockout mice display mild changes in skeletal muscle sarcomere structure, as well as in performance, particularly in the case of eccentric contractions [36, 37].

To gain further insight into the functions of *MARPs* in developing and mature striated muscle, we studied these genes in zebrafish, an animal model system with well-known advantages in genetic manipulation and *in vivo* analysis [38]. Here we report characterization of zebrafish *MARP* genes and proteins: their structure, comparison to the mammalian orthologs, expression profiles and localization during early development. In addition, we find differential upregulation of *MARP*s gene expression in striated muscle of adult zebrafish following endurance exercise. This study provides a foundation for further functional characterization of the MARP proteins in zebrafish development and stress response.

## Materials and methods

### Fish

The zebrafish (*Danio rerio*) AB strain was maintained on a 14 h light/10 h dark cycle at 28.5^°^C. Embryos obtained from wild-type fish were visually examined for proper development [39] and collected at several time points post fertilization. For *in situ* hybridization (ISH) experiments embryos older than 24 hpf were treated with 0.003% 1-phenyl 2-thiourea (PTU, Sigma-Aldrich, Merck KGaA, Darmstadt, Germany) to prevent pigmentation. Thirty two adult fish were used for exercise experiments and expression analysis. Zebrafish husbandry was performed under standard conditions in accordance with institutional and national ethical and animal welfare guidelines. Experiments were approved by the Veterinary Department, Darmstadt Regional Council, Germany and the Veterinary Directorate, Ministry of Agriculture, Forestry and Water Management, Republic of Serbia.

### Exercise protocol

Adult zebrafish (8 months old) were exercised in a 5L glass beaker (external diameter 170 mm) with a 60x10 mm stir bar, filled with 4L of fish water and placed on a magnetic stirrer, similarly to the Spinning Task described by Blazina *et al* [40]. Maximum number of fish in one beaker was 10. In order to adapt to the new conditions fish were pre-exercised for two days, 3 hours per day. Stirrer speed was adjusted to generate a 2 cm deep vortex. On the third day the rotation speed was increased to generate a 10 cm deep vortex and experiment was continued if 8 good swimmers remained. Fish that were able to swim continuously, while avoiding the vortex, were subjected to a regime consisting of two sets of 3 hours swimming, with 1 hour resting and feeding in between, for 5 consecutive days. Organs were harvested 3 hours after the last exercise bout. Skeletal muscles were sampled individually or pooled by two, while two hearts were pooled in each sample. Experimental groups contained at least 6 animals. Movie showing zebrafish swimming during exercise is given as supplemental file (S1 Movie), while detailed protocol is available at dx.doi.org/10.17504/protocols.io.sa2eage.

### RNA isolation and cDNA synthesis

Prior to RNA isolation embryos were mechanically dechorionated. Embryos and tissues of adult fish (axial skeletal muscles and whole hearts) were homogenized in Trizol (Life Technologies, Thermo Fisher Scientific, Waltham, Massachusetts, USA) using a Bullet Blender (Next Advance, Troy, New York, USA) or TissueLyser II (QIAGEN, Hilden, Germany). Total RNA from embryos was purified using RNeasy Mini Kit (Qiagen, Hilden, Germany), while RNA from adult tissues was isolated according to the standard manufacturer protocol for Trizol reagent. Isolated RNA was treated with Dnase I (Thermo Fisher Scientific, Waltham, Massachusetts, USA). Concentration and purity of RNA were determined by spectrophotometry using a NanoDrop 2000c (Thermo Fisher Scientific, Waltham, Massachusetts, USA). RNAs with A260/A280 ratio of 1.8–2.0 were used for downstream applications. Random hexamer-primed cDNA was synthesized by reverse transcription from 500 ng (for embryos) or 2 μg (for adult tissues) of total RNA using SuperScript III First-Strand Synthesis System (Invitrogen, Thermo Fisher Scientific, Waltham, Massachusetts, USA), iScript cDNA Synthesis Kit (Bio-Rad, Hercules, California, USA) or High Capacity cDNA Reverse Transcription Kit (Thermo Fisher Scientific, Waltham, Massachusetts, USA).

### Quantitative real-time PCR (qPCR)

qPCR was performed in technical triplicate for each sample on a CFX Connect Real-Time Detection System (Bio-Rad, Hercules, California, USA) or 7500 Real Time PCR System (Thermo Fisher Scientific, Waltham, Massachusetts, USA), using DyNAmo ColorFlash SYBR Green Master Mix (Thermo Fisher Scientific, Waltham, Massachusetts, USA) or Hot FIREPol EvaGreen qPCR Mix Plus (Solis BioDyne, Tartu, Estonia), respectively. The transcript of 60S ribosomal protein L13a gene (*rpl13a*) served as an internal reference to normalize the mRNA levels in different samples. The *rpl13a* mRNA expression level was not affected by the exercise or at any stage during development. The primers are listed in Table 1. Reaction conditions were as follows: initial denaturation at 95^°^C for 10 min, 40 cycles of denaturation at 95^°^C for 15 s, annealing and elongation at 60^°^C for 20 s when SYBR Green was used, and annealing at 60^°^C for 32 s followed by 20 s of elongation at 72^°^C when EvaGreen chemistry was used. Amplification was followed by the melting curve/dissociation analysis. The qPCR data were analyzed using the 2^(−ΔΔCt)^ method.

**Table 1:** List of primers used for *in situ* hybridization and quantitative PCR.

amplicon		forward primer 5′ - 3′	reverse primer 5′ - 3′	size (bp)
***in situ* hybridization**				
***ankrd1a***	AGGGTGGGAGAAAGTGCTTGT	CAAATGCTGAAAAGTTGTTCATCTG	902
***ankrd1b***	CTTCAAGCAACTGAAGTCCA	AATATGCAGGCTCATAATATCTCA	466
***ankrd2***	AGGCGTGAGATTGTTGATCTAGG	CTTTAGTGTCAAACTGCCACTGCT	664
***myod1***	CATTAACCCTCACTAAAGGGAATTCTACGACGACCCTTGCTT	TAATACGACTCACTATAGGGTTTCCAGCAGTGGATCAAAA	902
**quantitative PCR**				
***ankrd1a***	GAAGGGTGGGAGAAAGTGCT	TTTGGCTTCAGTTCACTTGG	
***ankrd1b***	CATCACAGGTGGAAACACAGA	CCGCTGAGAATGACTTCACC	
***ankrd2***	AGGGCATTACAGCCACTGAA	GTGCATCCCCAAGTGTTTGT	
***rpl13a***	TCTGGAGGACTGTAAGAGGTATGC	AGACGCACAATCTTGAGAGCAG	
***tnni2b*.*2***	AGGTGGACAGAGTTAATTACATGG	TCAGATCCTCAATCTCTTTGTCAC	
***tmod4***	CGCAACAGATGCTGAAATGTG	TTTCACCACACTGTTGATGCC	
***casq1a***	CTTCTTCAAGAGCAACAAATCC	GTTAATATCGTCTTCCCAGATCTC	
***casq1b***	ATAACACAGAGAATCCTGACCT	CCAGATACTCTCAGCATCATCC	
***tgfb2l***	CAGACACCTCCATATGCACAC	CACAGGTAAGGACAGTTCCC	
***mstnb***	CATGGCCACAGAACCTGACC	CCGGTCTCAGATGAACCCAG	
***col8a2***	AGGGTGAGTTTGTAATCTTGTGAC	CGTACTTCATCTGAGGCATAGG	
***lamc3***	CTAAAGATGCCAAAGCCTCCT	GAAGAAACCATGTCCTCCTCTG	
***cpt1b***	GCATTTCAGTTCACCGTCAC	AACACTGTTCTTAAAGCGGATGG	
***pfkma***	TCATGTCAGCAAAGGTAAGATCAC	AGTCTGTGCCAATAGTCATGTC	
***pdk2b***	GAATGAGCAACAGTTTGAAGGAG	AGAGTTTCCACAAATTCTGCGA	
***fbxo32***	CATTCAATCGCTTGGACTTCTG	TTGCTGATCATCGAGAACTTTCTG	
***dcn***	AAATTCCACTTGATACCACTCTCC	CCAAGATGAGCGTTTGGAGAC	
***aplnrb***	CATATTCTCTGATTCCCGTGCT	GAGCCAGGTTTCCAATGTAGAC	
***aplnra***	TAATGACTCTGGGTGTGACTACTC	GTTGCCGATATAAACATCTGCC	
***cs***	AGACCTCGTCCCTAAAGAACAG	CTCATTCCTCCATAAACCATGTCC	
***ppargc1a***	ACCCAGGTATGACAGCTATGAG	CTCGCCTCTCCTCTATTGCT	
***igf1***	TTATTTCAGCAAACCGACAGGA	GTTGTGCTCGTAGAGATCGT	
***il6r***	AGTGGATTTATAATGTGGACCCGA	CAGAAGGAGGATCTTGTCGAG	
***cxcl12b***	TTCCAAGTCATTGCCAAGCTG	CTTTAGAGATTCTCCGCTGTCC	
***igfbp2a***	CTAAACAGAGCCAGTGCCAG	CCACGATAGCCATTCACTGAC	
***casq2***	AACTTCCCATTGCTCATTCC	CTCGTCATCATTGGGTATCTC	
***sparc***	GAACTACAACATGTACATCTTCCC	CGACATCCTGCTCTTTGATCC	
***gys1***	AGAGTCAAAGTGATCTTCCATCC	AAACAGCCAAACCCAGACAG	
***ctrb1***	GATACAATGCTCCCGATACTC	ACACGATACCAACCAAAGTC	
***col1a1a***	GCTTCCAGTTCGAGTATGGC	GTGACACTGTATGTGAAGCGG	
***gpib***	CGCTTTCTACCAGCTCATCC	CAACAGAATCTTGTGGTGAAGG	
***lpl***	TCCATTATCAAGTGAAGGTCCA	GTTCAAAGTAGGCATAATGTAGGG	
***nppa***	CCAAGCTCAAGAGCTTGCTG	CTGCTTCCTCTCGGTCTCTG	
***pkmb***	CACACTCGGACCTGCTTCAC	ACGGACACTCTTGATGGTTTCAG	
***aldocb***	GAACCGCCGTCTTTACCGTC	ACACCTTTGTCAACCTTGATTCCT	
***ldha***	TGTTGGAATGGTAGGAATGGCTG	GCGGTCACACTGTAATCTTTATCC	

### *In situ* hybridization

Templates for synthesis of ISH probes were generated by PCR amplification using cDNA of 72 hpf embryos. Primer sets are given in Table 1. Amplicons were cloned into the pGEM-T easy vector (Promega, Madison, Wisconsin, USA) and verified by sequencing. Labeled RNA probes were synthesized using mMESSAGE mMACHINE SP6 or T7 kits (Thermo Fisher Scientific, Waltham, Massachusetts, USA) and digoxigenin RNA labeling nucleotide mix (Roche, Basel, Switzerland), with linearized plasmids as templates. Probes were analyzed by agarose gel electrophoresis and purified using RNA Clean and Concentrator-5 kit (Zymo Research, Irvine, California, USA). Probe for *myod1* was synthesized directly from PCR fragments amplified using primers containing T3 and T7 promoter sequences and cDNA of 48 hpf embryos. Whole mount ISH was performed according to the protocol of Thisse and Thisse [41]. Hybridization was carried out overnight at 67°C, with 300 ng of each probe. *myod1* probe was used as positive control (S1 Fig). Whole-mount embryo imaging was performed on a Nikon SMZ25 stereomicroscope (Nikon, Tokyo, Japan).

### Bioinformatics (protein sequence analysis, phylogeny and synteny)

The protein sequences of MARPs from different species were retrieved from the Ensembl database (http://www.ensembl.org/index.html). A list of accession numbers is shown in the S1 Table. Protein sequences were aligned using the Clustal Omega algorithm available on the EBI webserver [42]. Protein motifs for zebrafish, human and mouse MARPs were identified via the SMART database [43, 44]. PEST motifs and NLS sequences were identified using ePESTfind (http://emboss.bioinformatics.nl/cgi-bin/emboss/epestfind) and NLStradamus [45] web-based servers for sequence prediction, respectively. Analyses were conducted using the default parameters.

A phylogenetic tree was constructed using the maximum likelihood method of the PhyML algorithm (v3.0) [46], with bootstrapping value of 1000, via the ATGC webserver [47]. MARP protein sequences from zebrafish (*Danio rerio*), blind cave fish (*Astyanax mexicanus*), frog (*Xenopus tropicalis*), chicken (*Gallus gallus*), mouse (*Mus musculus*) and human (*Homo sapiens*) were analyzed. The genome assemblies are listed in S1 Table.

Synteny analysis of zebrafish and human genes was performed using Genomicus v87.01 genome browser synchronized with genomes from the Ensembl database [48].

### Nomenclature

Zebrafish gene and protein symbols are written according to 2018 ZFIN zebrafish nomenclature conventions. The zebrafish protein symbol is the same as the gene symbol, but non-italic and the first letter is uppercase. Human and mouse gene and protein symbols are written in accordance with HUGO and MGI nomenclatures, respectively. Human protein and gene symbols are both uppercase, and gene symbol is italic. Mouse protein symbol is uppercase, while gene symbols are italicized, with first letter uppercase.

### Statistical analysis

Developmental qPCR data were analyzed using one-way ANOVA followed by Tukey’s multiple comparison test. Expression levels in adult tissues were compared using the statistical t-test. Results were presented as mean ± SD, level of significance was *P*<0.05.

## Results

### Alignments, phylogenetic and synteny analysis of the zebrafish *MARP* genes

**Fig 1:**
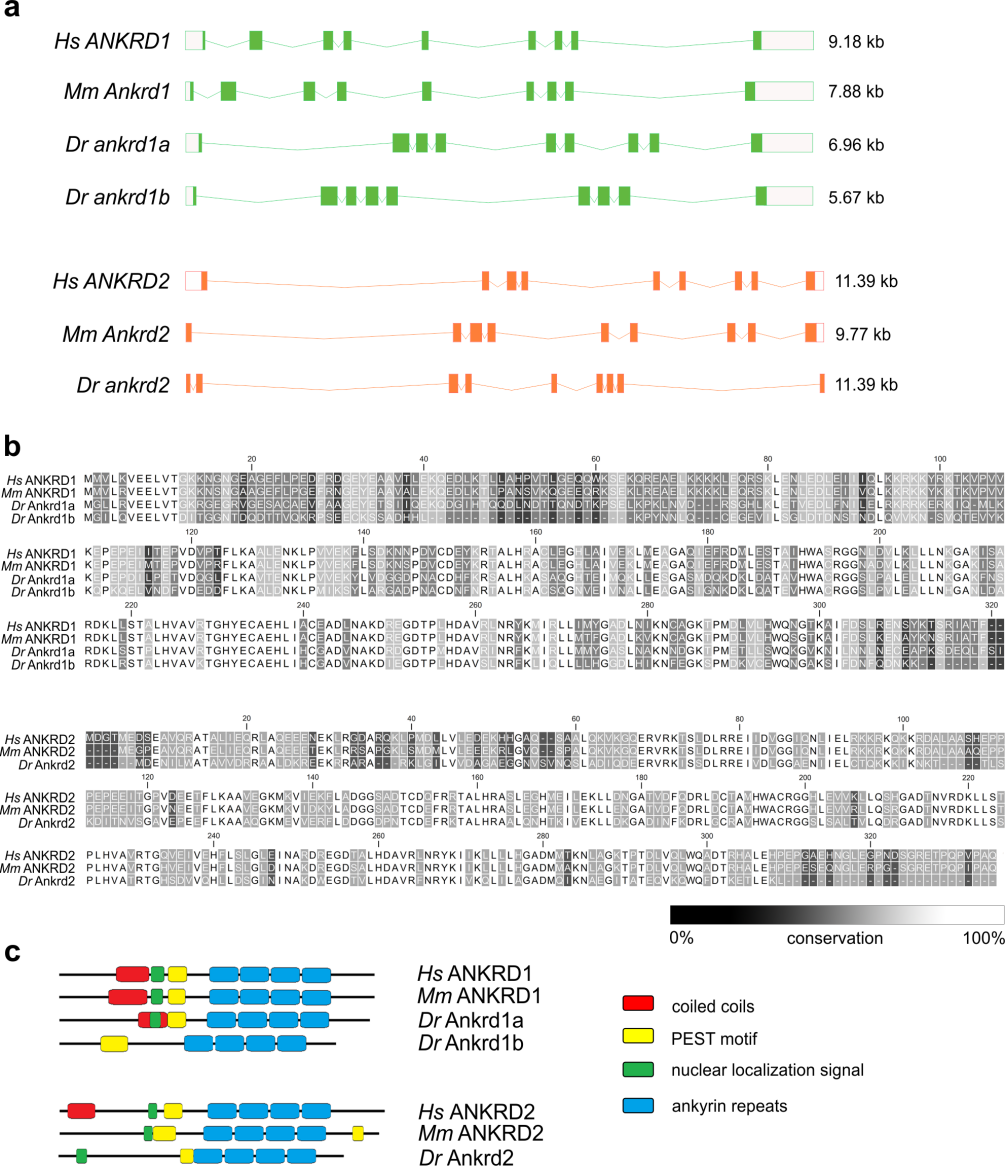
Gene organization, protein sequence alignment and domain structure of MARP family members from different species. (a) Exon-intron structure of human (*Hs*) *ANKRD1* and *ANKRD2* and their counterparts in mouse (*Mm*) and zebrafish (*Dr*). Exons (boxes) and introns (lines) are drawn to scale. White boxes indicate 5’ and 3’ UTRs. The numbers on the right indicate the length of the genomic region. (b) Amino acid sequence alignment of human, mouse and zebrafish proteins. (c) Schematic representation of the structural domains of human, mouse and zebrafish proteins. The predicted domains are indicated by colored boxes. Domain positions are listed in S2 Table.

The Ensembl database contains entries for three *MARP* genes in the zebrafish genome: *ankrd1a*, *ankrd1b* and *ankrd2*. First two are paralog genes, counterparts of the mammalian *ANKRD1/Ankrd1*. No gene corresponding to *ANKRD23/Ankrd23* was found in the zebrafish genome. The conserved structure of human, mouse and zebrafish *MARP* genes showing that all of them have nine exons is presented in Fig 1A.

The protein sequence alignment of human, mouse and zebrafish MARP orthologs shows substantial sequence similarities across species, particularly in the regions of ankyrin repeats (Fig 1B). BLAST comparison of zebrafish Ankrd1a, Ankrd1b and Ankrd2 with their human counterparts reveals 56%, 46% and 51% of identical amino acids (aa), respectively. Zebrafish Ankrd1a and Ankrd1b proteins show 47% identity. Zebrafish and mammalian MARP proteins also show similarities in other protein domains and motifs (Fig 1C). Ankyrin repeats, involved in protein-protein interactions, and PEST sequences, required for rapid intracellular proteolysis, are identified in all zebrafish MARPs. Protein oligomerization motif, coiled coils are predicted in Ankrd1a, but not in Ankrd1b and Ankrd2, while Ankrd1b lacks NLS. The position of these conserved functional domains within zebrafish MARP proteins is shown in S2 Table.

**Fig 2:**
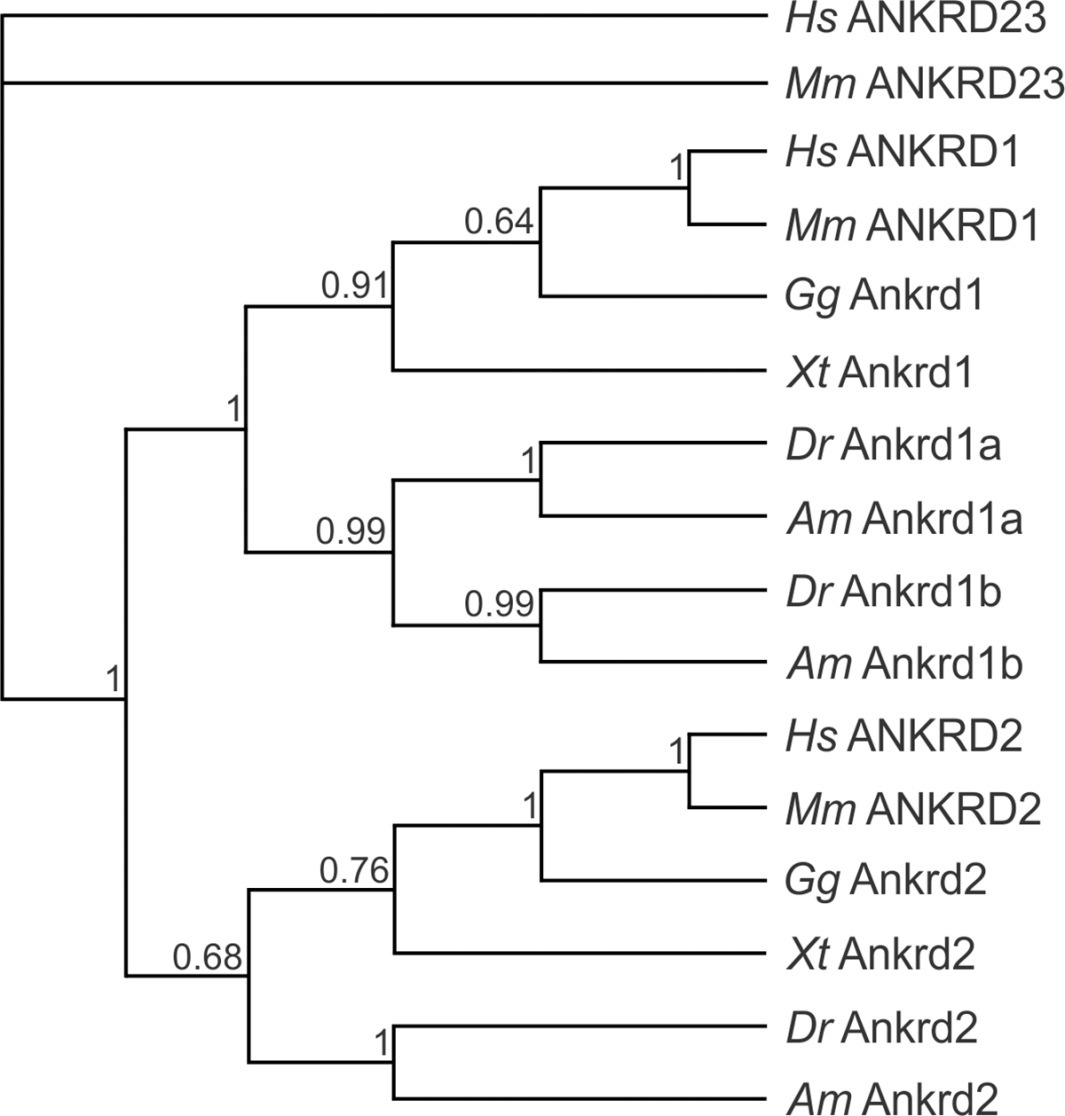
Phylogenetic tree of MARP homologs generated by PhyML algorithm. The GeneBank accession numbers of the sequences are listed in S1 Table. Species abbreviations: *Hs*, *Homo sapiens*; *Mm*, *Mus musculus*; *Gg*, *Gallus gallus*; *Xt*, *Xenopus tropicalis*; *Dr*, *Danio rerio*; *Am*, *Astyanax mexicanus*. Confidence of nodes is indicated by numbers on individual branches.

To investigate the evolutionary relationship, the phylogenetic tree based on Clustal Omega protein sequence alignment of MARP homologs was generated using the PhyML algorithm, with 1000 bootstraps (Fig 2). The tree topology segregates ANKRD1 and ANKRD2 homologs in two distinct groups. The Ankrd1b protein, present only in teleost, is closely related to Ankrd1a.

**Fig 3:**
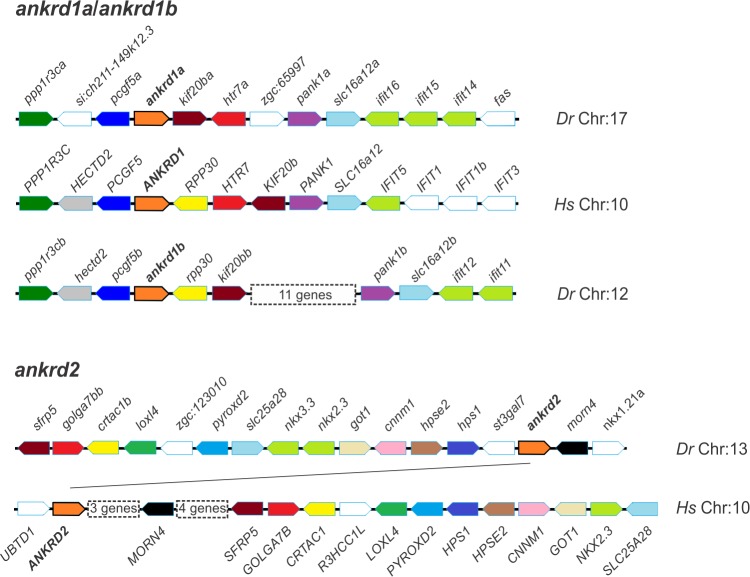
Synteny comparisons between human (*Hs*) and zebrafish (*Dr*) gene loci. *ANKRD1*/*ankrd1a/ankrd1b* (a) and *ANKRD2*/*ankrd2* (b) genes, depicted in orange polygons, are presented with nearest neighbors (colored polygons, orthologs have the same color). The transcriptional orientation of the gene is indicated by the angled end of each polygon corresponding to the 3´ end. Image style was adapted from Genomicus.

Syntenic analysis demonstrated that zebrafish *MARP* genes display a conserved genetic neighborhood with their human counterparts. Comparisons of chromosomal regions containing human *ANKRD1* and zebrafish *ankrd1a* and *ankrd1b* genes show that despite the rearrangements at the macrosyntenic level, neighboring genes and their homologs have kept their relative location throughout evolution (Fig 3). Regarding *ankrd2*, neighboring genes remained close but their orientation is inverted in comparison to human orthologs.

Overall, zebrafish MARPs are similar to their mammalian counterparts in terms of gene organization, primary protein sequence and identified key domains.

### Expression of the *MARP* genes in developing zebrafish

**Fig 4:**
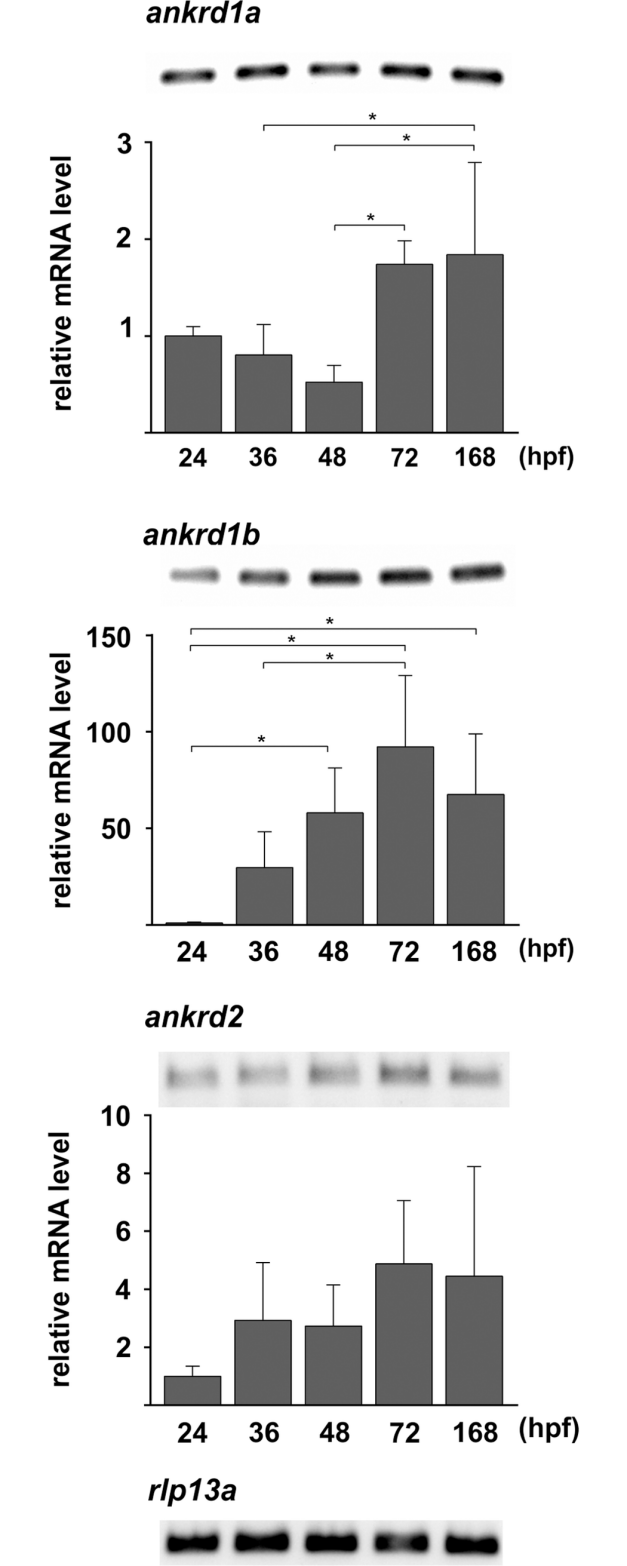
Quantification of zebrafish *MARP* transcripts during development. Expression levels of *ankrd1a*, *ankrd1b* and *ankrd2* at indicated time points after fertilization were obtained using qPCR. The housekeeping gene *rlp13a* served as internal reference. Data (mean ± SD) are combined from four biological replicates and normalized to the 24 hpf time point. * denotes *P*<0.05 in comparison to control group (by one-way ANOVA/Tukey’s multiple comparison test). Agarose gels showing qPCR products for *ankrd1a*, *ankrd1b*, *ankrd2* and *rlp13a* are also presented. Average Ct±SD values for *MARP* and reference (*rpl13a*) genes during zebrafish development at indicated time points are given in S3 Table.

We analyzed the temporal and spatial expression of *MARP* transcripts in the developing zebrafish embryos by qPCR and whole mount ISH. qPCR was performed using four independent batches of twenty embryos or larvae at each developmental stage tested. In general, although very low, the expression of all *MARPs* was increasing up to the seventh day of development (Fig 4 and S3 Table). A mild increase of *ankrd1a* expression was observed at 72 hpf (1.74-fold increase relative to 24 hpf time point), after which the levels did not change significantly up to 168 hpf. The most prominent change was shown for *ankrd1b* expression, with average of 92.18-fold increase in the first 72 hpf. Levels of *ankrd2* transcript were very low during first seven days of development and changes were not statistically significant. Among all *MARP* genes, *ankrd2* was the least expressed, as confirmed by the analysis of qPCR amplicons by agarose gel electrophoresis (Fig 4 and S3 Table).

**Fig 5:**
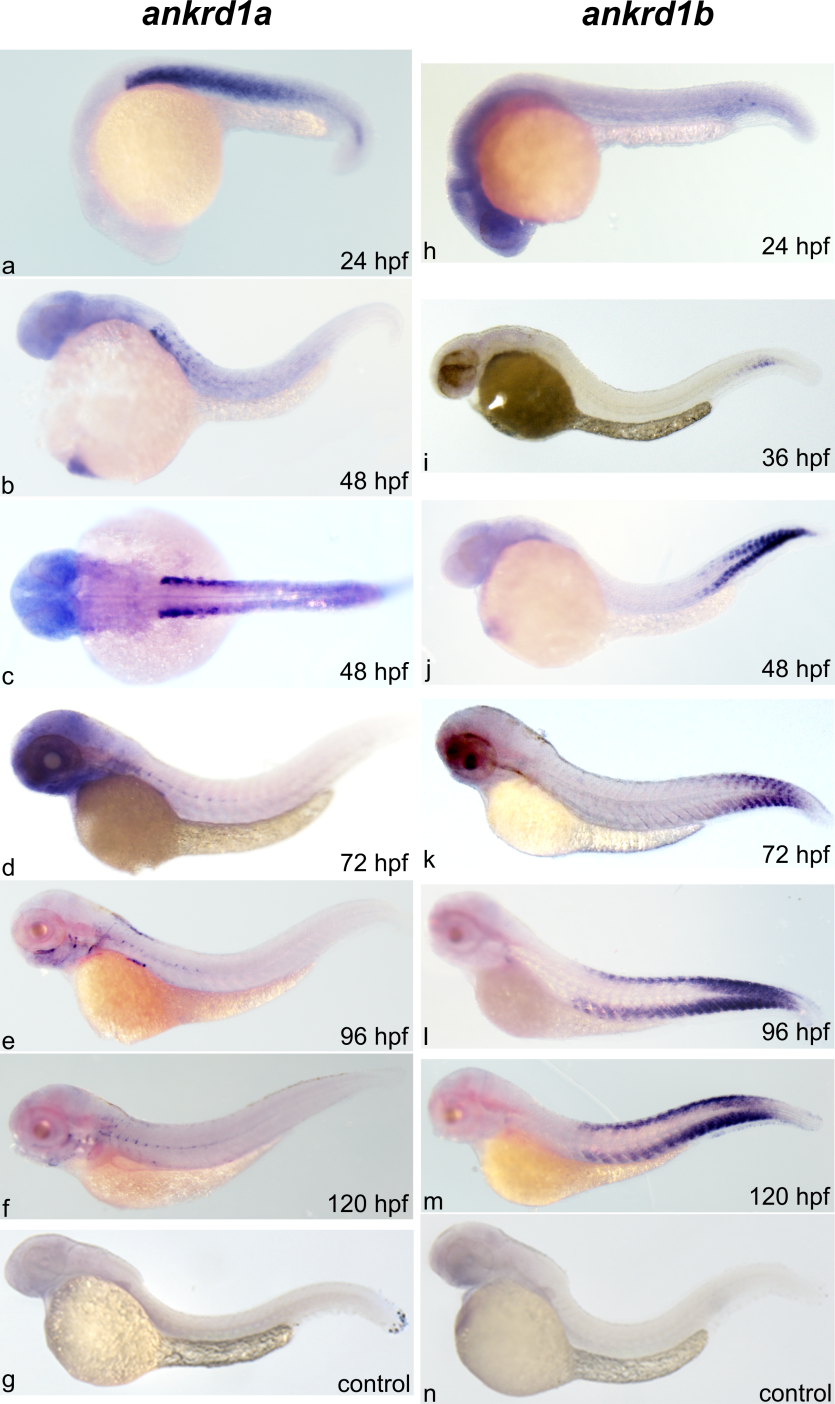
Spatiotemporal expression of zebrafish *ankrd1a* and *ankrd1b* during development, from 24 to 120 hpf. Representative images of whole mount ISH using probes detecting *ankrd1a* (a-f) and *ankrd1b* (h-m) transcripts at designated time points. Control staining for *ankrd1a* (g) and *ankrd1b* (n) was performed in 48 hpf embryos. Lateral and one dorsal (c) views are shown, anterior to the left.

As demonstrated by ISH, *ankrd1a* expression preceded that of the *ankrd1b* (Fig 5). First transcripts of *ankrd1a* were observed at 24 hpf, in the ventral part of the developing somites. At later stages expression of *ankrd1a* was more spatially restricted, concentrated in the apex of the chevron-shaped somites. Conversely, *ankrd1b* expression was first observed at 36 hpf in the tail somites. As development progresses, *ankrd1b* gene expression expands caudally, being more pronounced in the most ventral and dorsal parts of the somites. We were not able to detect any signal for *ankrd2* even after prolonged staining, consistent with qPCR results.

In conclusion, during development, *ankrd1a* and *ankrd1b* are expressed in somites, in a non-overlapping pattern, and at relatively low levels.

### Endurance exercise differentially upregulates expression of the *MARP* genes in adult zebrafish heart and skeletal muscle

**Fig 6:**
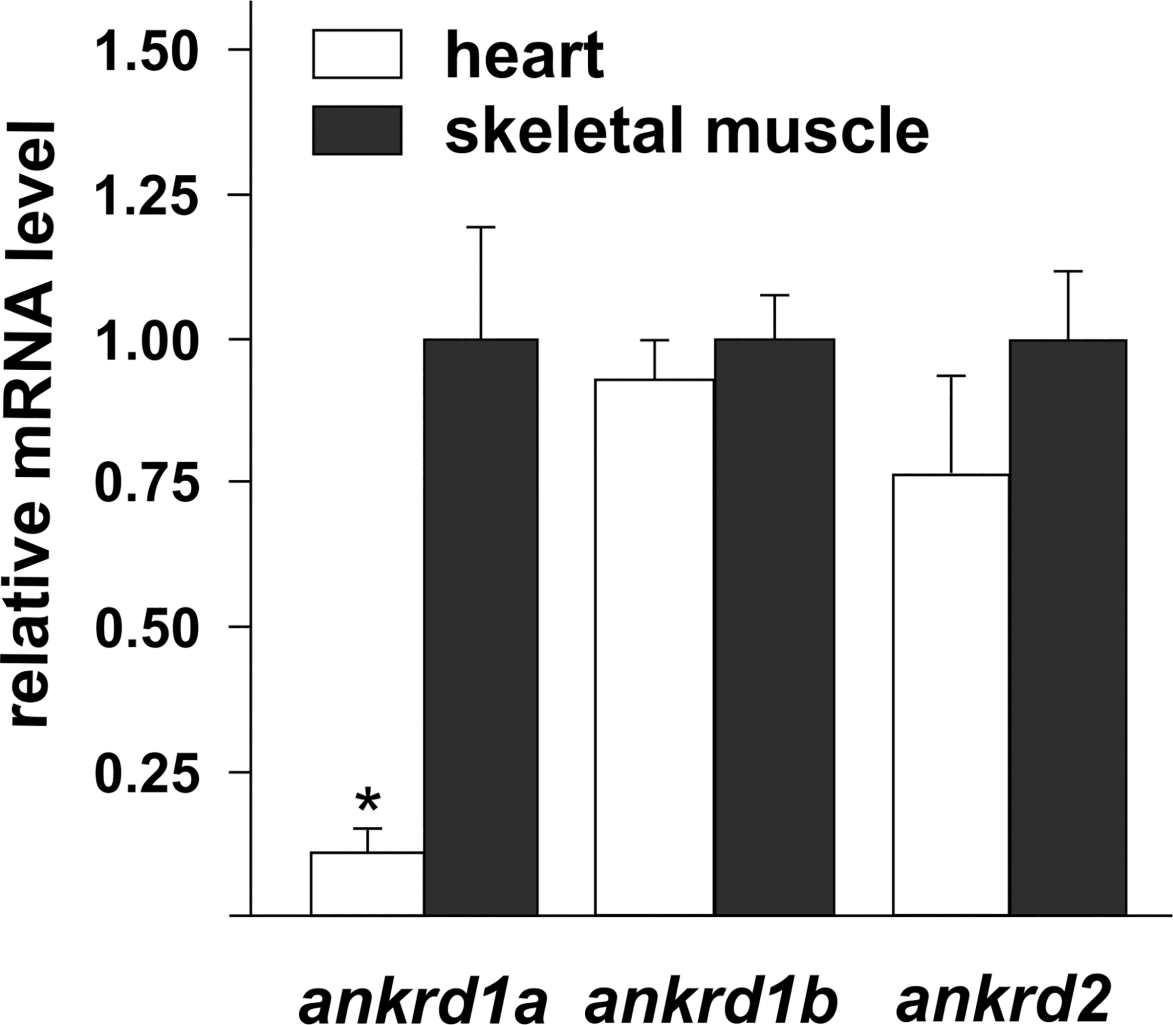
Expression of the zebrafish *MARP* genes in adult heart and skeletal muscle. Quantification of *ankrd1a*, *ankrd1b* and *ankrd2* expression was done by qPCR, using mRNA isolated from the hearts of 8 fish, pooled in 4 groups and the skeletal muscles of 4 fish. Relative level of transcripts in adult heart is normalized to the transcript level in the skeletal muscle, set as 1. Bars represent the mean ± SD. * denotes *P*<0.05 in comparison to control group (by t-test). Average Ct±SD values for *MARP* and reference (*rpl13a*) genes in adult zebrafish heart and skeletal muscle are given in S4 Table.

Since human *ANKRD1* and *ANKRD2* genes are differentially expressed in cardiac and skeletal muscles, we investigated relative expression of zebrafish genes in these two organs in adult animals. Under basal conditions, all *MARP* genes are expressed at low levels (S4 Table), *ankrd1a* being the most abundant. There is more *ankrd1a* transcript in the skeletal muscle, compared to the heart, while *ankrd1b* and *ankrd2* show no significant difference in distribution between analyzed tissues (Fig 6).

**Fig 7:**
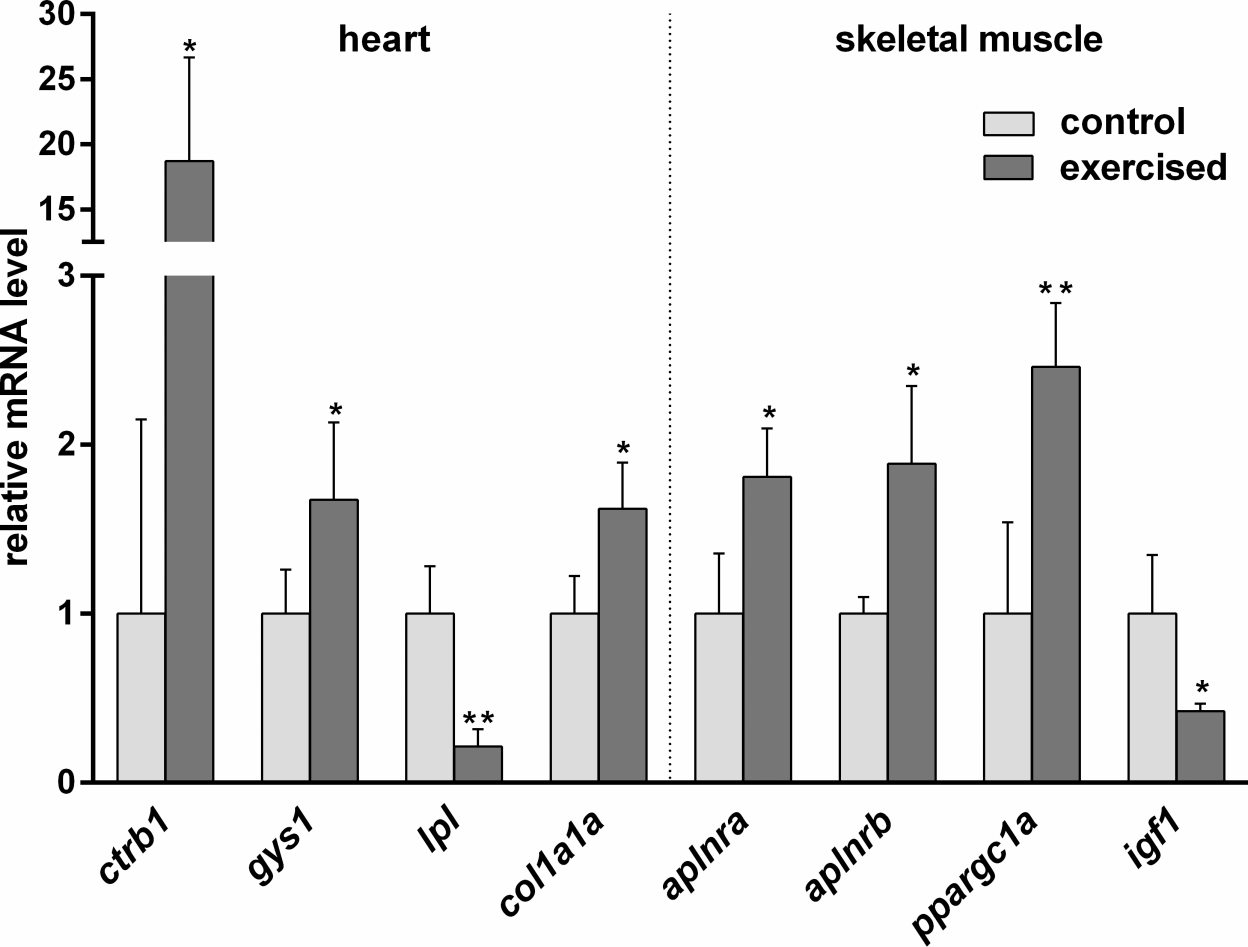
Effects of swimming exercise on heart and skeletal muscle genes expression. mRNA expression levels of *chymotrypsinogen B1* (*ctrb1*), *glycogen synthase 1* (*gys1*), *lipoprotein lipase* (*lpl*) and *type I collagen*, *alpha 1a* (*col1a1a*) in heart, and *apelin receptor a* (*aplnra*), *apelin receptor b* (*aplnrb*), *peroxisome proliferator-activated receptor gamma*, *coactivator 1 alpha* (*ppargc1a*) and *insulin-like growth factor 1* (*igf1*) in skeletal muscle of exercised fish expressed as a fold change over non-exercised controls set to 1. Bars represent the mean ± SD. * denotes *P*<0.05 and ** denotes *P*<0.005 in comparison to control group (by t-test). Fold change ± SD values for all tested cardiac and skeletal muscle genes of trained and control adult zebrafish are given in S5 Table.

In order to analyze the responsiveness of zebrafish *ankrd1a*, *ankrd1b* and *ankrd2* genes to increased muscle activity we quantified their mRNA levels in whole hearts and skeletal muscle after one week of endurance exercise. To validate that employed exercise protocol is able to cause changes in cardiac and skeletal muscle gene expression comparable to those observed after tunnel swimming, we measured expression of exercise-responsive genes listed in S5 Table. Majority of these genes were selected based on their reported altered expression in zebrafish after tunnel swimming of varying duration [49–52]. Additionally, several genes from mammalian model organisms and humans were included in the analysis [53–55]. Among the tested genes, four in the heart (*col1a1a*, *lpl*, *gys1* and *ctrb1*) and four in skeletal muscle (*ppargc1a*, *aplnra*, *aplnrb* and *igf1*) showed significant change in expression after exercise (Fig 7). These results recapitulate known effects of swim tunnel exercise and support the expected activation of muscles.

**Fig 8:**
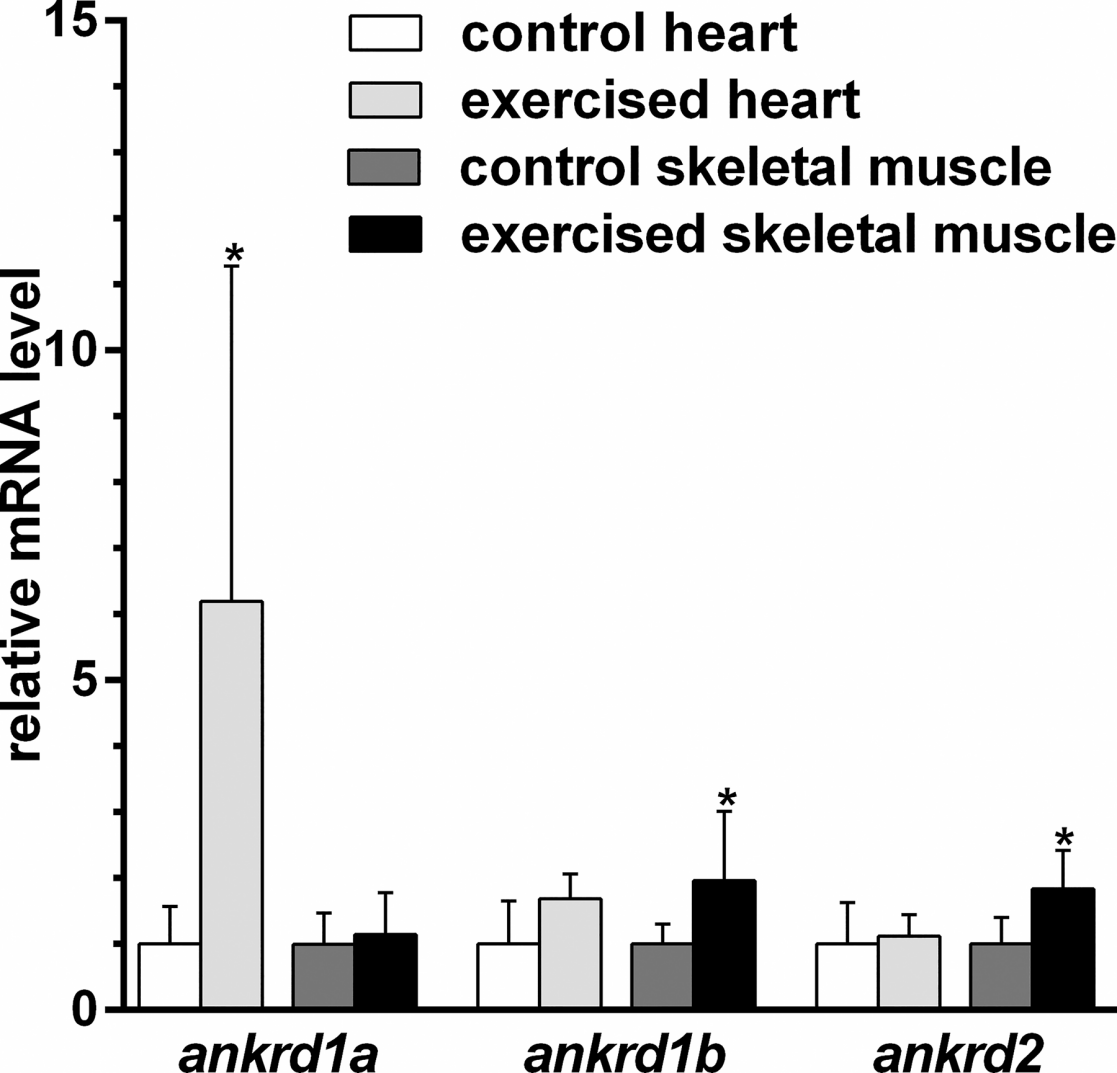
Zebrafish *MARP* genes are differentially upregulated in adult heart and in skeletal muscle after endurance exercise. Adult fish were subjected to endurance exercise for one week. Relative expression of *MARP* genes in zebrafish heart and skeletal muscle is expressed as fold change to non-exercised controls. Bars represent the mean ± SD. * denotes *P*<0.05 in comparison to control group (by t-test).

Endurance exercise caused significant increase in *ankrd1a* mRNA level (fold change 6.19 ± 5.08 compared to non-exercised control fish) in adult hearts (Fig 8). No change was observed for *ankrd1b* and *ankrd2* transcripts levels in the heart. However, a slight upregulation of *ankrd1b* and *ankrd2* expression in skeletal muscles was detected (1.97±1.05 and 1.84±0.58, respectively) in exercised animals. These results indicate that, like in mammals, zebrafish MARPs are responsive to increased load imposed on striated muscle.

## Discussion

In this study we investigated protein structure, evolutionary conservation, spatiotemporal expression profiles and responsiveness to increased muscle activity of zebrafish *MARP* genes *ankrd1a*, *ankrd1b* and *ankrd2*. As suggested by the phylogenetic tree topology, the former two are paralog genes, orthologous to mammalian *ANKRD1*/*Ankrd1*, most probably originating from a duplication event in the teleost lineage. This notion is further supported by the synteny analysis which shows similarities shared by the two loci in regard to the genomic context.

The primary sequences of MARP proteins in all compared taxa are modestly conserved, with identical residue percentage being around 50% in all pairwise comparisons. Protein sequences of the two zebrafish paralogs, Ankrd1a and Ankrd1b, show more divergence in the N-terminal region, with the former being more similar to its orthologs in other species. Despite showing 50% aa identity to human and mouse orthologs, there are notable differences in the N-terminal region of the zebrafish Ankrd2 protein.

Most of the protein sequence identity is shared between the ankyrin repeats, a motif all MARPs have in common, which is indispensable for their interactions with other proteins [1]. The coiled-coil domain, essential for homo- and heterodimerization of MARP proteins in antiparallel fashion [56, 57], is present in Ankrd1a, but missing from Ankrd1b and Ankrd2. The NLS is predicted in Ankrd1a and Ankrd2, but not in Ankrd1b. The identified structural similarities of zebrafish MARP family members to their relatively distant mammalian counterparts suggests that their function is conserved. Further experimental work is needed to characterize zebrafish MARPs at the protein level in cardiac and skeletal muscle, including their relative amounts and subcellular distribution.

Profiles of basal expression of *ankrd1a*, *ankrd1b* and *ankrd2* genes in developing and adult zebrafish mostly differ from those observed for their mammalian counterparts. During cardiogenesis, murine *Ankrd1* transcript and protein are expressed specifically in the myocardium and slightly stronger in the atrium than in the ventricle [3, 58]. Human ANKRD1 was found to be strongly expressed in the fetal heart, diffusely distributed throughout the atria and ventricles, while ANKRD2 was detectable at trace levels [4, 59]. During the first five days of zebrafish development, none of the *MARP* transcripts were detected in the heart by *in situ* hybridization, indicating no or very low expression, undetectable by this method. Expression of mouse fetal *Ankrd2* transcript is restricted to skeletal muscle [10], in contrast to *Ankrd1*, which is not detected in this organ during development [3]. In humans, both ANKRD1 and ANKRD2 were detected in fetal skeletal muscle [4]. Zebrafish *ankrd1a* and *ankrd1b* transcripts were only found in developing axial muscles. Absence of *ankrd2* expression suggests no important role during early zebrafish development. Our results are in accordance with the data in the EMBO Expression Atlas on *ankrd1a* and *ankrd1b* expression [60]. Gene expression analysis by qPCR revealed that *ankrd1b* mRNA levels increased during development, peaking at 72 hpf. Similarly, *ankrd1a* expression was detected during embryonic stages, observing a significant increase in larvae at 72 and 168 hpf. Interestingly, our ISH analysis shows that at larval stages the number of *ankrd1a* expressing cells is reduced. A possible explanation is that, despite being present in lower number than at earlier stages, these cells express higher levels of *ankrd1a*. On the other hand, contribution of other larval cells expressing low amounts of *ankrd1a*, not detectable by ISH staining, cannot be excluded. Differential spatial expression patterns suggest that *ankrd1a* and *ankrd1b* expression may be regulated by trunk and tail muscle specific regulators. Development of zebrafish trunk and tail muscle domains is controlled by different mechanisms. Trunk domains are established via Nodal signaling, whereas the tail domain requires BMP during early development [61]. A spatial expression pattern similar to that of *ankrd1a* and *ankrd1b* was observed for myosin heavy chain isoforms coded by *fmyhc1*.*2* and *fmyhc2*.*1* whose differential expression in trunk and tail, respectively, is coordinated by retinoic acid and Wnt signaling [62]. Use of gene knockout, overexpressing and reporter zebrafish lines will help in deciphering regulatory mechanisms of *ankrd1a* and *ankrd1b* expression and their distinct functions during zebrafish development.

Expression of MARPs in adult mammalian skeletal muscle and the heart is well documented [3–5, 59, 63]. In humans, ANKRD1 and ANKRD2 were detected mostly in the heart and skeletal muscles, respectively. Contrary to human homologs, adult zebrafish *MARP* genes have low expression under basal conditions. While *ankrd1b* and *ankrd2* transcripts are equally distributed between heart and skeletal muscles, *ankrd1a* is preferentially expressed in skeletal muscle, similarly to avian *ANKRD1* gene [64]. Low levels of *ankrd1a* and *ankrd1b* expression in the adult zebrafish heart, detected by qPCR, is in line with results obtained during transcriptome analysis of zebrafish genes homologous to dilated cardiomyopathy-associated human genes [65].

Mammalian MARPs are known to be upregulated by various stress stimuli. Endurance exercise and eccentric contractions [9, 11, 13, 66], hypertrophic overload of skeletal muscle [67], chronic immobilization of leg muscles in a stretched position [10, 16], submaximal exhaustive exercise [14] and fatiguing jumping exercise [12] all increase expression of mammalian *ANKRD1*/*Ankrd1* and/or *ANKRD2*/*Ankrd2*. Here we demonstrated evolutionary conservation of *MARPs* responsiveness to endurance exercise which differentially upregulated *ankrd1a* in heart and *ankrd1b* and *ankrd2* in skeletal muscle. Differential response of *ankrd1a* and *ankrd1b* paralogs to endurance exercise suggests their non-redundant functions. Generally, after gene duplication, one of the paralog genes is often lost from the genome due to redundancy [68], while if duplicated genes acquire non-redundant functions, both are likely to be retained [69]. In the case of *ankrd1a* and *ankrd1b* it is possible that a functional specialization in the cardiac and skeletal muscle occurred.

Alterations in expression of cardiac and skeletal muscle genes after exercise were mostly studied in fish subjected to linear tunnel swimming [49–51, 70, 71] and their transcriptomic response to endurance exercise was determined by microarray analysis [49, 51]. The exercise method used in this study is similar to published set-ups with varying swimming conditions, employed to study motor coordination [40] and swim performance [72]. To demonstrate that our swimming protocol caused the activation of striated muscle in adult zebrafish, we analyzed the expression of selected exercise responsive genes from different functional categories: muscle growth and development, muscle contraction, extracellular matrix, protein synthesis and degradation, metabolism, and myokines [49–51]. Recapitulation of known aspects of tunnel swimming training suggests that exercise method used in this study is a valuable tool for investigating muscle response to increased load in zebrafish, as an affordable alternative to costly swim tunnels. It is worth noting that exercise as short as the one used in this study induces the expression of *MARPs*, demonstrating their early responsiveness in zebrafish striated muscle to increased activity. Since response of mammalian *MARP* genes vary depending on the type, intensity and duration of the exercise and recovery period, expression studies after various forms and regimes of exercise are needed. The zebrafish model provides a tool to asses MARPs function in stressed striated muscle by virtue of the protocol we used, which is adjustable to facilitate various exercise regimes.

Differential upregulation of zebrafish *MARP* genes in cardiac and skeletal muscles after one week of endurance exercise training suggests their possible muscle-type specific role in physiological remodeling and may be a reflection of differences between mechanisms of cardiac and skeletal muscles adaptation to increased workload. It was already demonstrated that endurance exercise differentially stimulates development of heart and axial muscle in zebrafish [70]. The phenotype of axial muscles was shifted towards a slow aerobic, while heart muscle gained a faster phenotype, but does not become more aerobic.

Despite differences in primary structure, gene number and expression patterns between zebrafish and mammalian MARPs, their responsiveness to increased muscle activity is encouraging for including zebrafish as a model organism for further functional studies of these genes in mature muscles. It is worth mentioning that *ankrd1a* has been identified in recent studies as an early response gene in regeneration of injured zebrafish heart [73–75]. These and our data point to remodeling of skeletal muscles and cardiac regeneration as processes in which the role of zebrafish MARPs warrants further investigation.

## Conclusions

The expression profiles of *ankrd1a* and *ankrd1b* indicate an active role in the development of somites, while upregulation of gene expression of all zebrafish *MARPs* after relatively short endurance exercise suggests that their function in response to increased activity in striated muscles is conserved. This initial study provides a foundation from which the zebrafish could be established as a novel model organism for further functional studies of *MARPs* in mature striated muscle.

## Supporting information

S1 Table Accession numbers of MARP proteins from different species and genome assemblies. (DOCX)

S2 Table Domain positions in zebrafish MARP proteins. (DOCX)

S3 Table Average Ct±SD values for *MARP* and reference (*rpl13a*) genes during zebrafish development at indicated time points. (DOCX)

S4 Table Average Ct±SD values for *MARP* and reference (*rpl13a*) genes in adult zebrafish heart and skeletal muscle. (DOCX)

S5 Table Expression levels of selected zebrafish genes after one week of endurance exercise. (DOCX)

S1 Fig Detection of *myod1* transcript in 48 hpf embryos by ISH. (TIF)

S1 Movie A Movie showing zebrafish swimming during exercise. (AVI)
