# Supplementary material for: Characterization of zebrafish (Danio rerio) muscle ankyrin repeat proteins reveals their conserved response to endurance exercise
Source: PLoS One. 2018 Sep 25;13(9):e0204312. doi: 10.1371/journal.pone.0204312 (PMC6155536; doi:10.1371/journal.pone.0204312)
Supplement: S1 Table — (DOCX) [file pone.0204312.s001.docx]

S1 Table. Accession numbers of MARP proteins from different species and genome assemblies

| **Organism** | **Protein** | **Accession number** | **Genome assembly** |
| --- | --- | --- | --- |
| *Danio rerio* | Ankrd1a | ENSDARP00000098001.3 | GRCz10 |
|  | Ankrd1b | ENSDARP00000097889.2 |  |
|  | Ankrd2 | ENSDARP00000069315.4 |  |
| *Homo sapiens* | ANKRD1 | ENSP00000360762.3 | GRCh38.p7 |
|  | ANKRD2 | ENSP00000359689.1 |  |
| *Mus musculus* | ANKRD1 | ENSMUSP00000025718.8 | GRCm38.p5 |
|  | ANKRD2 | ENSMUSP00000026172.2 |  |
| *Gallus gallus* | ANKRD1 | ENSGALP00000010477.2 | GCA_000002315.3 |
|  | ANKRD2 | ENSGALP00000012178.5 |  |
| *Xenopus tropicalis* | Ankrd1 | ENSXETP00000047089.3 | GCA_000004195.1 |
|  | Ankrd2 | ENSXETP00000007445.3 |  |
| *Astyanax mexicanus* | Ankrd1a | ENSAMXP00000009303.1 | GCA_000372685.1 |
|  | Ankrd1b | ENSAMXP00000007927.1 |  |
|  | Ankrd2 | ENSAMXP00000002269.1 |  |
