# Supplementary material for: Characterization of zebrafish (Danio rerio) muscle ankyrin repeat proteins reveals their conserved response to endurance exercise
Source: PLoS One. 2018 Sep 25;13(9):e0204312. doi: 10.1371/journal.pone.0204312 (PMC6155536; doi:10.1371/journal.pone.0204312)
Supplement: S2 Table — (DOCX) [file pone.0204312.s002.docx]

S2 Table. Domain positions in zebrafish MARP proteins

|  | **Ankrd1a** | **Ankrd1b** | **Ankrd2^a^** |
| --- | --- | --- | --- |
| ENSEMBL transcript ID | ENSDART00000112959.3 | ENSDART00000113810.2 | ENSDART00000074829.4 |
| UniProt | E7F095 | F1QQ98 | F1Q6Y2 |
| Lenght (aa) | 317 | 283 | 296 |
| Ankyrin repeats | 144-177  181-210  214-243  247-276 | 124-153  157-186  190-219  223-252 | 108-137  141-170  174-203  207-236  240-269 |
| NLS | 91-99 | / | 20-31 |
| PEST | 104-122 | 41-70 | 128-143 |
| Coiled coil | 77-105 | / | / |

^a^Ankrd2 transcript ENSDART00000146027.2 coding for 236 aa protein was not taken into analysis
