# Supplementary material for: Characterization of zebrafish (Danio rerio) muscle ankyrin repeat proteins reveals their conserved response to endurance exercise
Source: PLoS One. 2018 Sep 25;13(9):e0204312. doi: 10.1371/journal.pone.0204312 (PMC6155536; doi:10.1371/journal.pone.0204312)
Supplement: S3 Table — (DOCX) [file pone.0204312.s003.docx]

S3 Table. Average Ct±SD values for *MARP* and reference (*rpl13a*) genes during zebrafish development at indicated time points.

|  | 24hpf | 36hpf | 48hpf | 72hpf | 168hpf |
| --- | --- | --- | --- | --- | --- |
| ***ankrd1a*** | 31.94±0.33 | 32.42±0.68 | 32.94±0.73 | 32.07±0.46 | 32.82±1.35 |
| ***ankrd1b*** | 35.28±0.52 | 30.63±0.75 | 29.44±0.70 | 29.48±0.69 | 30.76±1.09 |
| ***ankrd2*** | 37.13±0.87 | 35.96±0.92 | 35.83±1.03 | 35.68±1.20 | 37.04±1.13 |
| ***rpl13a*** | 21.98±0.45 | 22.07±0.29 | 22.00±0.39 | 22.90±0.49 | 23.50±0.37 |

hpf-hours post fertilization
