# Supplementary material for: Characterization of zebrafish (Danio rerio) muscle ankyrin repeat proteins reveals their conserved response to endurance exercise
Source: PLoS One. 2018 Sep 25;13(9):e0204312. doi: 10.1371/journal.pone.0204312 (PMC6155536; doi:10.1371/journal.pone.0204312)
Supplement: S4 Table — (DOCX) [file pone.0204312.s004.docx]

S4 Table. Average Ct±SD values for *MARP* and reference (*rpl13a*) genes in adult zebrafish heart and skeletal muscle.

|  | **heart** | **skeletal muscle** |
| --- | --- | --- |
| ***ankrd1a*** | 24.63±1.44 | 21.09±1.11 |
| ***ankrd1b*** | 28.47±0.27 | 28.09±0.62 |
| ***ankrd2*** | 31.20±0.83 | 30.56±0.72 |
| ***rpl13a*** | 17.47±0.52 | 17.14±0.58 |
