# Supplementary material for: Characterization of zebrafish (Danio rerio) muscle ankyrin repeat proteins reveals their conserved response to endurance exercise
Source: PLoS One. 2018 Sep 25;13(9):e0204312. doi: 10.1371/journal.pone.0204312 (PMC6155536; doi:10.1371/journal.pone.0204312)
Supplement: S5 Table — (DOCX) [file pone.0204312.s005.docx]

S5 Table. Expression level of selected zebrafish genes after one week of endurance exercise

| gene symbol | gene name | Fold change ± SD | | *P* value | function | ref. |
| --- | --- | --- | --- | --- | --- | --- |
|  |  | control | exercised |  |  |  |
| **heart** | | | | | | |
| *cxcl12b* | chemokine (C-X-C motif) ligand | 1.00 ± 0.31 | 0.75 ± 0.23 | 0.24 | muscle growth and development | [51] |
| *igfbp2a* | insulin-like growth factor binding protein | 1.00 ± 0.21 | 1.12 ± 0.69 | 0.75 |  | [51] |
| *casq2* | calsequestrin 2 | 1.00 ± 0.36 | 1.05 ± 0.15 | 0.79 | muscle contraction | [51] |
| *sparc* | secreted protein, acidic, cysteine-rich (osteonectin) | 1.00 ± 0.24 | 1.56 ± 0.52 | 0.10 | extracellular matrix | [51] |
| ***col1a1a*** | collagen, type I, alpha 1a | 1.00 ± 0.22 | 1.62 ± 0.27 | 0.04 |  | [51] |
| *nppa* | natriuretic peptide A | 1.00 ± 0.21 | 0.94 ± 0.25 | 0.73 | natriuresis | [54] |
| *gpib* | glucose-6-phosphate isomerase b | 1.00 ± 0.55 | 0.61 ± 0.08 | 0.21 | carbohydrate metabolism | [51] |
| ***gys1*** | glycogen synthase 1 | 1.00 ± 0.26 | 1.67 ± 0.46 | 0.04 |  | [51] |
| *pkmb* | pyruvate kinase M1/2b | 1.00 ± 0.62 | 1.81 ± 1.65 | 0.39 | glucose metabolism | [53] |
| *aldocb* | aldolase C, fructose-bisphosphate, b | 1.00 ± 0.26 | 1.23 ± 0.35 | 0.34 |  | [55] |
| *ldha* | lactate dehydrogenase A4 | 1.00 ± 0.27 | 1.17 ± 0.34 | 0.47 |  | [53, 55] |
| ***lpl*** | lipoprotein lipase | 1.00 ± 0.28 | 0.21 ± 0.10 | **0.002** | lipid metabolism | [51] |
| ***ctrb1*** | chymotrypsinogen B1 | 1.00 ± 1.15 | 18.70 ± 7.98 | **0.006** | protein synthesis and degradation | [51] |
| **skeletal muscle** | | | | | | |
| *tnni2b.2* | troponin I type 2b | 1.00 ± 0.44 | 1.20 ± 0.64 | 0.63 | muscle contraction | [49] |
| *tmod4* | tropomodulin | 1.00 ± 0.38 | 0.62 ± 0.17 | 0.12 |  | [49] |
| *casq1a* | calsequestrin 1a | 1.00 ± 0.39 | 0.58 ± 0.16 | 0.09 |  | [49] |
| *casq1b* | calsequestrin 1b | 1.00 ± 0.39 | 0.55 ± 0.16 | 0.07 |  | [49] |
| *tgfb2l* | transforming growth factor, beta 2, like | 1.00 ± 0.37 | 0.74 ± 0.15 | 0.24 | muscle growth and development | [49] |
| *mstnb* | myostatin b | 1.00 ± 0.58 | 0.57 ± 0.33 | 0.25 |  | [49] |
| *col8a2* | collagen | 1.00 ± 0.49 | 1.29 ± 0.61 | 0.49 | extracellular matrix | [49] |
| *lamc3* | laminin, gamma 3 | 1.00 ± 0.31 | 1.18 ± 0.58 | 0.61 |  | [49] |
| *cpt1b* | carnitine palmitoyltransferase | 1.00 ± 0.35 | 0.93 ± 0.51 | 0.82 | metabolism | [49] |
| *pfkma* | phosphofructokinase | 1.00 ± 0.42 | 0.86 ± 0.43 | 0.67 |  | [49] |
| *cs* | citrate synthase | 1.00 ± 0.26 | 0.85 ± 0.29 | 0.47 |  | [52,53] |
| ***ppargc1a*** | peroxisome proliferator-activated receptor gamma, coactivator 1 alpha | 1.00 ± 0.54 | 2.46 ± 0.37 | **0.004** |  | [52] |
| *pdk2b* | pyruvate dehydrogenase kinase, isozyme 2b | 1.00 ± 0.46 | 0.52 ± 0.47 | 0.19 | protein synthesis and degradation | [49] |
| *fbxo32* | F-box protein 32 | 1.00 ± 0.63 | 1.02 ± 1.01 | 0.97 |  | [49] |
| ***aplnra*** | apelin receptor b | 1.00 ± 0.35 | 1.81 ± 0.29 | **0.01** | myokines | [50] |
| ***aplnrb*** | apelin receptor b | 1.00 ± 0.10 | 1.89 ± 0.46 | **0.02** |  | [50] |
| ***igf1*** | insulin-like growth factor 1 | 1.00 ± 0.35 | 0.42 ± 0.04 | **0.04** |  | [50] |
| *dcn* | decorin | 1.00 ± 0.25 | 1.02 ± 0.28 | 0.90 |  | [50] |
| *il6r* | interleukin 6 receptor | 1.00 ± 0.06 | 1.4 ± 0.67 | 0.28 |  | [50] |
