# Supplementary figures and images for: Characterization of zebrafish (Danio rerio) muscle ankyrin repeat proteins reveals their conserved response to endurance exercise
Source: PLoS One. 2018 Sep 25;13(9):e0204312. doi: 10.1371/journal.pone.0204312 (PMC6155536; doi:10.1371/journal.pone.0204312)

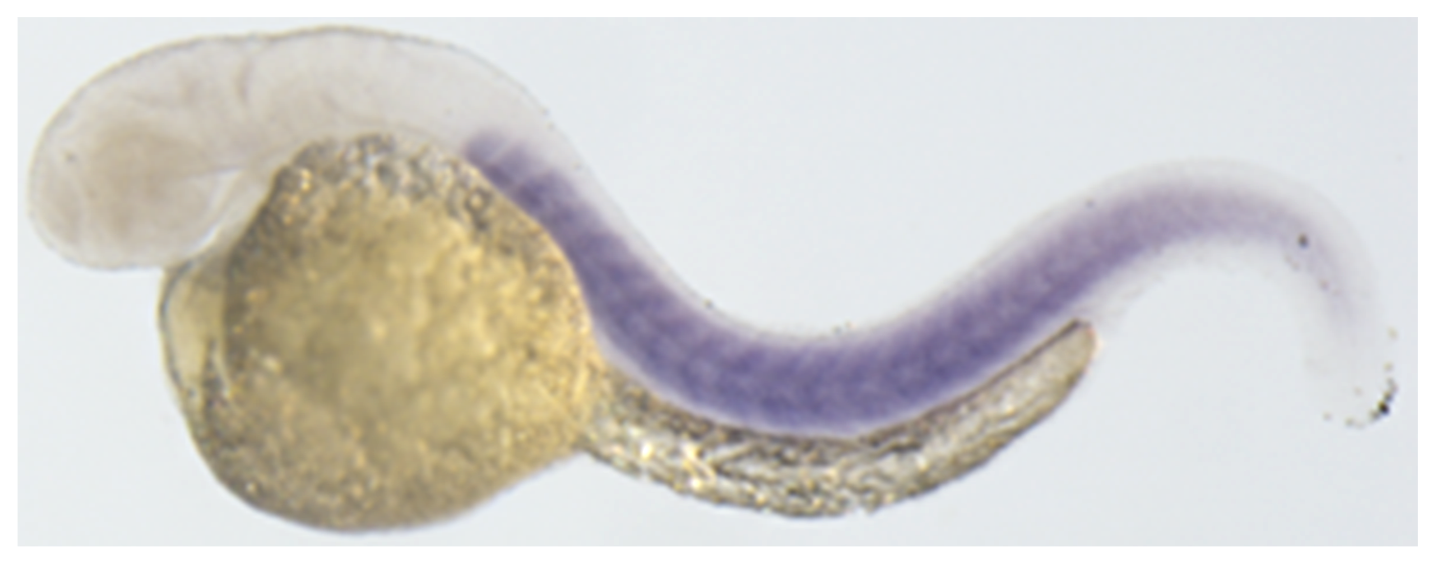

Supplement: S1 Fig — (TIF) [file pone.0204312.s006.tif]
